# Special Collection of *Health Equity*: Introduction

**DOI:** 10.1089/heq.2021.29010.intro

**Published:** 2021-09-21

**Authors:** Gail C. Christopher

**Affiliations:** Executive Director, National Collaborative for Health Equity, Washington, District of Columbia, USA.

A major health crisis such as a pandemic can generate an unprecedented opportunity for societal transformation. Clean drinking water systems, sewers, and public parks are among the innovations that grew from previous pandemics. Coronavirus disease 2019 (COVID-19) now compels us to innovate. The current public discourse focuses on new investments in infrastructure and includes a newly coined term, human infrastructure.

Human infrastructure refers to our realization that systems of support and care for children, elderly, and care providers make daily living possible and help to sustain our democracy by making our economy viable. However, our capacity for truly caring for one another in the United States is impeded by our failure to face and to finally eliminate the false ideology of a hierarchy of human value.

This failure to face and rid our cultural ethos of racism and permission to devalue groups of people based on physical characteristics is a major fault line in the soul of America. We must address this challenge. America must galvanize the public will to come together and do the work of ending racism and its protracted devastating generational consequences. These destructive consequences were evidenced in the shocking disparities and inequities in COVID-19 diagnoses and deaths.

The Centers for Disease Control and Prevention (CDC) reported that African Americans were twice as likely as White Americans to die from COVID-19 in 2020. In Washington, DC, the nation's capital, the government reported that Black people are 45% of the population and comprised 76% of the deaths from COVID-19. This pattern of racial disparities was seen in cities and states across the country. Excess deaths among people of color, Native Americans, Latinos, and African Americans as well as among immigrant and refugee population groups are a result of increased exposure to the virus, high levels of pre-existing chronic conditions, and barriers to care resulting from historic and present-day discrimination. All of these factors are symptoms of the true driver of racial health inequities—racism—and our societal refusal to eliminate it.

This special collection of *Health Equity* is devoted to the Truth Racial Healing and Transformation (TRHT) movement across America. TRHT is a United States adaptation of the globally recognized Truth and Reconciliation (TRC) process. It is adapted to address the entrenched centuries old nature of the racism challenge faced by the United States, the ever-expanding diversity of the U.S. population, and the urgent need for transforming opportunity structures and systems.

Launched in 2016 by the W.K. Kellogg Foundation in collaboration with several other local philanthropies, TRHT is currently in place on college campuses, in communities, and in public health organizations across America. TRHT can be a vital catalytic force for postpandemic transformation in 2021 and beyond. By providing a comprehensive framework and strategic process for engaging diverse representatives from multiple sectors, TRHT helps communities move beyond polarization toward a shared vision for and pathway to create a transformed present and future in which racism has been faced, jettisoned, and its harms redressed and healed. Now that over 200 local jurisdictions and the director of the CDC have declared that racism is a public health crisis, there is added momentum for efforts to eliminate racism and its consequences. TRHT posits that our efforts must be collective, and they must be guided by shared visioning, reimagining an America that has faced, reconciled its past, and generated a transformed present and future.

This special collection brings together important documents to frame the issues around truth, racial healing, and transformation (TRHT). They provide thoughtful analysis from nonprofit leaders, experts in public policy, and seasoned foundation professionals. Starting with the USTRHT Funders Briefing from earlier this year, as well as a recent interview on the Impacts of Social Equity on Health from the National Academy of Public Administration, there is also an international perspective with testimony from Gail Christopher for the Commission on Security and Cooperation in Europe provided in Helsinki in July 2019. An earlier article from the fall of 2017 from the National Civic Review grounds this collection in discussion which began more than 4 years ago. Together these presentations provide high-level, context, justification, and a road map for moving forward.

## Abbreviations Used

CDC: Centers for Disease Control and Prevention
COVID-19: coronavirus disease 2019
TRC: Truth and Reconciliation
TRHT: Truth Racial Healing and Transformation

